# Diaqua­bis(*N*,*N*-diethyl­nicotinamide-κ*N* ^1^)bis­(4-formyl­benzoato-κ*O*)cobalt(II)

**DOI:** 10.1107/S1600536809008265

**Published:** 2009-03-11

**Authors:** Mustafa Sertçelik, Barış Tercan, Ertan Şahin, Hacali Necefoğlu, Tuncer Hökelek

**Affiliations:** a Kafkas University, Department of Chemistry, 63100 Kars, Turkey; b Karabük University, Department of Physics, 78050 Karabük, Turkey; c Atatürk University, Department of Chemistry, 22240 Erzurum, Turkey; d Hacettepe University, Department of Physics, 06800 Beytepe, Ankara, Turkey

## Abstract

In the crystal structure of the title Co^II^ complex, [Co(C_8_H_5_O_3_)_2_(C_10_H_14_N_2_O)_2_(H_2_O)_2_], the metal centre is located on an inversion center and is coordinated by two 4-formyl­benzoate (FOB), two diethyl­nicotinamide (DENA) ligands and two water mol­ecules in a slightly distorted CoO_4_N_2_ octa­hedral geometry. In the crystal structure, O—H⋯O hydrogen bonds link the mol­ecules into infinite chains. π–π contacts between the parallel pyridine rings of neighboring DENA ligands [centroid–centroid distance = 3.652 (3) Å] further stabilize the crystal structure.

## Related literature

For general background, see: Antolini *et al.* (1982 ▶); Bigoli *et al.* (1972 ▶); Nadzhafov *et al.* (1981 ▶); Shnulin *et al.* (1981 ▶). For related structures, see: Hökelek *et al.* (1995 ▶, 1997 ▶, 2007 ▶, 2008 ▶); Hökelek & Necefoğlu (1996 ▶, 1997 ▶, 2007 ▶); Sertçelik *et al.* (2009*a* ▶, 2009*b* ▶).
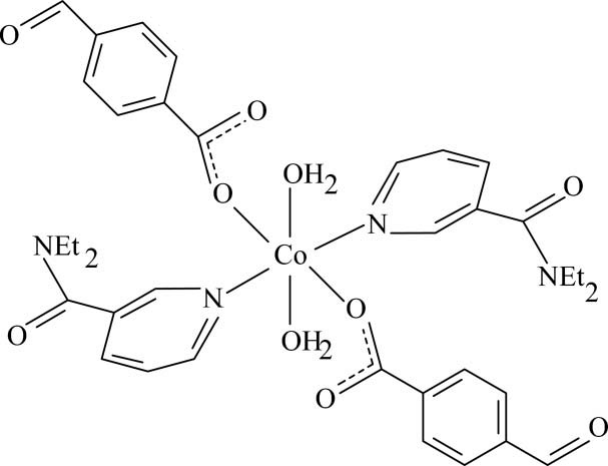


## Experimental

### 

#### Crystal data

[Co(C_8_H_5_O_3_)_2_(C_10_H_14_N_2_O)_2_(H_2_O)_2_]
*M* *_r_* = 749.67
Triclinic, 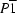

*a* = 7.2962 (2) Å
*b* = 8.6863 (3) Å
*c* = 15.9453 (5) Å
α = 85.433 (2)°
β = 78.608 (3)°
γ = 68.022 (2)°
*V* = 918.64 (5) Å^3^
*Z* = 1
Mo *K*α radiation
μ = 0.53 mm^−1^
*T* = 294 K
0.35 × 0.25 × 0.15 mm

#### Data collection

Rigaku R-AXIS RAPID-S diffractometer
Absorption correction: multi-scan (Blessing, 1995 ▶) *T*_min_ = 0.853, *T*_max_ = 0.926
19487 measured reflections
3755 independent reflections
3016 reflections with *I* > 2σ(*I*)
*R*_int_ = 0.074

#### Refinement

*R*[*F* ^2^ > 2σ(*F* ^2^)] = 0.069
*wR*(*F* ^2^) = 0.195
*S* = 1.07
3755 reflections
246 parameters
3 restraints
H atoms treated by a mixture of independent and constrained refinement
Δρ_max_ = 1.02 e Å^−3^
Δρ_min_ = −0.33 e Å^−3^

### 

Data collection: *CrystalClear* (Rigaku/MSC, 2005 ▶); cell refinement: *CrystalClear*; data reduction: *CrystalClear*; program(s) used to solve structure: *SHELXS97* (Sheldrick, 2008 ▶); program(s) used to refine structure: *SHELXL97* (Sheldrick, 2008 ▶); molecular graphics: *Mercury* (Macrae *et al.*, 2006 ▶); software used to prepare material for publication: *WinGX* (Farrugia, 1999 ▶).

## Supplementary Material

Crystal structure: contains datablocks I, global. DOI: 10.1107/S1600536809008265/xu2489sup1.cif

Structure factors: contains datablocks I. DOI: 10.1107/S1600536809008265/xu2489Isup2.hkl

Additional supplementary materials:  crystallographic information; 3D view; checkCIF report

## Figures and Tables

**Table 1:** Selected bond lengths (Å)

Co1—O1	2.088 (3)
Co1—O5	2.121 (3)
Co1—N1	2.163 (3)

**Table 2:** Hydrogen-bond geometry (Å, °)

*D*—H⋯*A*	*D*—H	H⋯*A*	*D*⋯*A*	*D*—H⋯*A*
O5—H51⋯O4^i^	0.94 (4)	1.86 (4)	2.787 (5)	174 (6)
O5—H52⋯O2^ii^	0.92 (2)	1.73 (4)	2.646 (5)	168 (6)

Symmetry codes: (i) 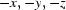; (ii) 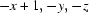.

**Fig. 1.**
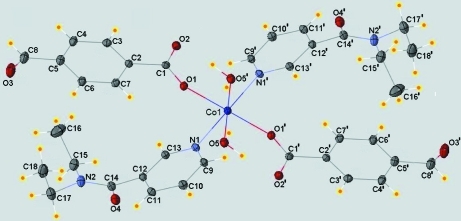
The molecular structure of the title molecule with the atom-numbering scheme. Displacement ellipsoids are drawn at the 50% probability level. Hydrogen bonds are shown as dashed lines. Primed atoms are generated by the symmetry operator (1 -x, -y, -z).

**Fig. 2.**
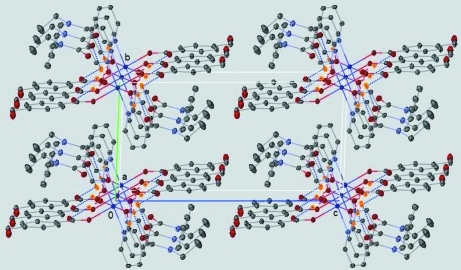
A partial packing diagram of (I) viewed down the a axis, showing hydrogen bonds (dotted lines) linking the molecules into chains, where b and c axes are vertical and horizontal, respectively. H atoms not involved in hydrogen bonding are omitted.

## supplementary crystallographic information

### Comment

Transition metal complexes with biochemically active ligands frequently show interesting physical and/or chemical properties, as a result they may find applications in biological systems (Antolini *et al.*, 1982). The structural functions and coordination relationships of the arylcarboxylate ion in transition metal complexes of benzoic acid derivatives change depending on the nature and position of the substituent groups on the benzene ring, the nature of the additional ligand molecule or solvent, and the medium of the synthesis (Nadzhafov *et al.*, 1981; Shnulin *et al.*, 1981). The nicotinic acid derivative *N*,*N*-diethylnicotinamide (DENA) is an important respiratory stimulant (Bigoli *et al.*, 1972).

The structure determination of the title compound, (I), a cobalt complex with two formylbenzoate (FOB), two diethylnicotinamide (DENA) ligands and two water molecules, was undertaken in order to determine the properties of the ligands and also to compare the results obtained with those reported previously.

Compound (I) is a monomeric complex, with the Co atom on a centre of symmetry. It contains two FOB, two DENA ligands and two water molecules (Fig. 1). All ligands are monodentate. The four O atoms (O1, O5, and the symmetry-related atoms, O1', O5') in the equatorial plane around the Co atom form a slightly distorted square-planar arrangement, while the slightly distorted octahedral coordination is completed by the two N atoms of the DENA ligands (N1, N1') in the axial positions (Table 1 and Fig. 1).

The near equality of the C1—O1 [1.262 (5) Å] and C1—O2 [1.257 (5) Å] bonds in the carboxylate group indicates a delocalized bonding arrangement, rather than localized single and double bonds, and may be compared with the corresponding distances: 1.263 (4) and 1.249 (4) Å in [Ni(DENA)_2_(C_8_H_5_O_3_)_2_(H_2_O)_2_], (II), (Sertçelik *et al.*, 2009*a*), 1.262 (3) and 1.249 (3) Å in [Mn(DENA)_2_ (C_8_H_5_O_3_)_2_(H_2_O)_2_], (III), (Sertçelik *et al.*, 2009*b*), 1.256 (6) and 1.245 (6) Å in [Mn(DENA)_2_(C_7_H_4_ClO_2_)_2_(H_2_O)_2_], (IV), (Hökelek *et al.*, 2008), 1.265 (6) and 1.275 (6) Å in [Mn(C_9_H_10_NO_2_)_2_(H_2_O)_4_]. 2(H_2_O), (V), Hökelek & Necefoğlu, 2007), 1.260 (4) and 1.252 (4) Å in [Zn(DENA)_2_(C_7_H_4_FO_2_)_2_(H_2_O)_2_], (VI), (Hökelek *et al.*, 2007), 1.259 (9) and 1.273 (9) Å in Cu_2_(DENA)_2_(C_6_H_5_COO)_4_, (VII), (Hökelek *et al.*, 1995), 1.279 (4) and 1.246 (4) Å in [Zn_2_(DENA)_2_(C_7_H_5_O_3_)_4_]. 2H_2_O, (VIII), (Hökelek & Necefoğlu, 1996), 1.251 (6) and 1.254 (7) Å in [Co(DENA)_2_(C_7_H_5_O_3_)_2_(H_2_O)_2_], (IX), (Hökelek & Necefoğlu, 1997), 1.278 (3) and 1.246 (3) Å in [Cu(DENA)_2_(C_7_H_4_NO_4_)_2_(H_2_O)_2_], (*X*), (Hökelek *et al.*, 1997). This may be due to the intermolecular O—H···O hydrogen bonding of the carboxylate O atoms (Table 2). In (I), the average Co—O bond length is 2.105 (3) Å and the Co atom is displaced out of the least-squares plane of the carboxylate group (O1/C1/O2) by 0.768 (1) Å. The dihedral angle between the planar carboxylate group and the benzene ring A (C2—C7) is 4.02 (35)°, while that between rings A and B (N1/C9—C13) is 79.61 (14)°.

In the crystal structure, intermolecular O—H···O hydrogen bonds (Table 1) link the molecules into infinite chains (Fig. 2), in which they may be effective in the stabilization of the structure. The π-π contact between the DENA rings, *Cg*1—*Cg*1^i^ [symmetry code: (i) 2 - *x*, 1 - *y*, -*z*, where *Cg*1 is centroid of the ring B (N1/C9—C13)] may further stabilize the structure, with centroid-centroid distance of 3.652 (3) Å.

### Experimental

The title compound was prepared by the reaction of CoSO_4_.H_2_O (1.73 g, 10 mmol) in H_2_O (50 ml) and DENA (3.56 g, 20 mmol) in H_2_O (15 ml) with sodium 4-formylbenzoate (3.44 g, 20 mmol) in H_2_O (50 ml). The mixture was filtered and set aside to crystallize at ambient temperature for several days, giving red single crystals.

### Refinement

H atoms of water molecule and formyl group were located in difference Fourier maps and refined isotropically, with restrains of O5—H51 = 0.94 (4), O5—H52 = 0.92 (2) Å, H51—O5—H52 = 105 (3)° and *U*_iso_(H) = 0.09 (2) and 0.075 (18) Å^2^ (for H_2_O); C8—H81 = 1.04 (6) Å and *U*_iso_(H) = 0.086 (19) Å^2^ (for formyl group). The remaining H atoms were positioned geometrically with C—H = 0.93, 0.97 and 0.96 Å, for aromatic, methylene and methyl H atoms and constrained to ride on their parent atoms, with *U*_iso_(H) = *xU*_eq_(C), where *x* = 1.5 for methyl H atoms, and *x* = 1.2 for all other H atoms.

### Crystal data

**Table:**

[Co(C_8_H_5_O_3_)_2_(C_10_H_14_N_2_O)_2_(H_2_O)_2_]	*Z* = 1
*M**_r_* = 749.67	*F*(000) = 393
Triclinic, *P*1	*D*_x_ = 1.355 Mg m^−^^3^
Hall symbol: -P 1	Mo *K*α radiation, λ = 0.71073 Å
*a* = 7.2962 (2) Å	Cell parameters from 4172 reflections
*b* = 8.6863 (3) Å	θ = 2.5–26.4°
*c* = 15.9453 (5) Å	µ = 0.53 mm^−^^1^
α = 85.433 (2)°	*T* = 294 K
β = 78.608 (3)°	Prism, red
γ = 68.022 (2)°	0.35 × 0.25 × 0.15 mm
*V* = 918.64 (5) Å^3^	

### Data collection

**Table:**

Rigaku R-AXIS RAPID-S diffractometer	3755 independent reflections
Radiation source: fine-focus sealed tube	3016 reflections with *I* > 2σ(*I*)
graphite	*R*_int_ = 0.074
ω scans	θ_max_ = 26.4°, θ_min_ = 2.5°
Absorption correction: multi-scan (Blessing, 1995)	*h* = −9→9
*T*_min_ = 0.853, *T*_max_ = 0.926	*k* = −10→10
19487 measured reflections	*l* = −19→19

### Refinement

**Table:**

Refinement on *F*^2^	Primary atom site location: structure-invariant direct methods
Least-squares matrix: full	Secondary atom site location: difference Fourier map
*R*[*F*^2^ > 2σ(*F*^2^)] = 0.069	Hydrogen site location: inferred from neighbouring sites
*wR*(*F*^2^) = 0.195	H atoms treated by a mixture of independent and constrained refinement
*S* = 1.07	*w* = 1/[σ^2^(*F*_o_^2^) + (0.0999*P*)^2^ + 0.5936*P*] where *P* = (*F*_o_^2^ + 2*F*_c_^2^)/3
3755 reflections	(Δ/σ)_max_ < 0.001
246 parameters	Δρ_max_ = 1.02 e Å^−^^3^
3 restraints	Δρ_min_ = −0.33 e Å^−^^3^

### Special details

**Table:**

Geometry. All e.s.d.'s (except the e.s.d. in the dihedral angle between two l.s. planes) are estimated using the full covariance matrix. The cell e.s.d.'s are taken into account individually in the estimation of e.s.d.'s in distances, angles and torsion angles; correlations between e.s.d.'s in cell parameters are only used when they are defined by crystal symmetry. An approximate (isotropic) treatment of cell e.s.d.'s is used for estimating e.s.d.'s involving l.s. planes.
Refinement. Refinement of *F*^2^ against ALL reflections. The weighted *R*-factor *wR* and goodness of fit *S* are based on *F*^2^, conventional *R*-factors *R* are based on *F*, with *F* set to zero for negative *F*^2^. The threshold expression of *F*^2^ > σ(*F*^2^) is used only for calculating *R*-factors(gt) *etc*. and is not relevant to the choice of reflections for refinement. *R*-factors based on *F*^2^ are statistically about twice as large as those based on *F*, and *R*- factors based on ALL data will be even larger.

### Fractional atomic coordinates and isotropic or equivalent isotropic displacement parameters (Å^2^)

**Table:**

	*x*	*y*	*z*	*U*_iso_*/*U*_eq_	
Co1	0.5000	0.0000	0.0000	0.0386 (3)	
O1	0.4778 (5)	−0.1144 (4)	−0.10591 (18)	0.0473 (7)	
O2	0.7579 (5)	−0.1290 (4)	−0.1979 (2)	0.0555 (8)	
O3	0.0578 (8)	−0.1907 (7)	−0.4559 (3)	0.0958 (15)	
O4	−0.2309 (5)	0.3315 (4)	−0.1246 (2)	0.0585 (9)	
O5	0.2256 (5)	−0.0208 (4)	0.0630 (2)	0.0506 (8)	
H51	0.224 (10)	−0.126 (4)	0.080 (3)	0.09 (2)*	
H52	0.214 (9)	0.033 (5)	0.113 (2)	0.075 (18)*	
N1	0.3188 (5)	0.2363 (4)	−0.0485 (2)	0.0419 (8)	
N2	−0.1116 (7)	0.4197 (6)	−0.2512 (3)	0.0609 (11)	
C1	0.5813 (6)	−0.1268 (5)	−0.1802 (3)	0.0422 (9)	
C2	0.4825 (6)	−0.1361 (5)	−0.2530 (3)	0.0418 (9)	
C3	0.5824 (7)	−0.1374 (6)	−0.3370 (3)	0.0496 (11)	
H3	0.7097	−0.1317	−0.3480	0.060*	
C4	0.4938 (8)	−0.1471 (7)	−0.4037 (3)	0.0570 (12)	
H4	0.5613	−0.1474	−0.4596	0.068*	
C5	0.3035 (8)	−0.1566 (6)	−0.3882 (3)	0.0546 (11)	
C6	0.2016 (7)	−0.1530 (6)	−0.3045 (3)	0.0510 (11)	
H6	0.0741	−0.1583	−0.2934	0.061*	
C7	0.2906 (7)	−0.1417 (6)	−0.2383 (3)	0.0463 (10)	
H7	0.2213	−0.1377	−0.1824	0.056*	
C8	0.2121 (10)	−0.1698 (9)	−0.4608 (4)	0.0750 (16)	
H81	0.294 (9)	−0.169 (7)	−0.522 (4)	0.086 (19)*	
C9	0.3415 (6)	0.3797 (5)	−0.0385 (3)	0.0439 (10)	
H9	0.4366	0.3784	−0.0070	0.053*	
C10	0.2310 (7)	0.5285 (6)	−0.0724 (3)	0.0494 (10)	
H10	0.2502	0.6255	−0.0636	0.059*	
C11	0.0905 (7)	0.5306 (5)	−0.1200 (3)	0.0478 (10)	
H11	0.0157	0.6287	−0.1449	0.057*	
C12	0.0637 (6)	0.3839 (5)	−0.1299 (3)	0.0426 (9)	
C13	0.1798 (6)	0.2402 (5)	−0.0936 (3)	0.0419 (9)	
H13	0.1613	0.1421	−0.1005	0.050*	
C14	−0.1028 (7)	0.3743 (6)	−0.1700 (3)	0.0468 (10)	
C15	0.0391 (10)	0.4689 (8)	−0.3107 (3)	0.0732 (16)	
H15A	0.1301	0.4851	−0.2785	0.088*	
H15B	−0.0288	0.5745	−0.3370	0.088*	
C16	0.1577 (13)	0.3470 (13)	−0.3789 (6)	0.136 (4)	
H16A	0.2601	0.3820	−0.4123	0.203*	
H16B	0.0709	0.3392	−0.4151	0.203*	
H16C	0.2193	0.2404	−0.3536	0.203*	
C17	−0.2911 (10)	0.4212 (8)	−0.2844 (4)	0.0754 (16)	
H17A	−0.4114	0.4706	−0.2420	0.090*	
H17B	−0.3049	0.4888	−0.3359	0.090*	
C18	−0.2719 (12)	0.2533 (8)	−0.3035 (5)	0.097 (2)	
H18A	−0.3828	0.2589	−0.3290	0.146*	
H18B	−0.2721	0.1894	−0.2515	0.146*	
H18C	−0.1483	0.2014	−0.3424	0.146*	

### Atomic displacement parameters (Å^2^)

**Table:**

	*U*^11^	*U*^22^	*U*^33^	*U*^12^	*U*^13^	*U*^23^
Co1	0.0376 (5)	0.0409 (5)	0.0385 (5)	−0.0145 (3)	−0.0104 (3)	0.0022 (3)
O1	0.0497 (17)	0.0557 (18)	0.0396 (16)	−0.0214 (15)	−0.0118 (13)	0.0004 (13)
O2	0.0445 (18)	0.069 (2)	0.0525 (18)	−0.0191 (16)	−0.0098 (14)	−0.0061 (16)
O3	0.100 (3)	0.148 (5)	0.068 (3)	−0.070 (3)	−0.032 (2)	0.002 (3)
O4	0.0547 (19)	0.070 (2)	0.062 (2)	−0.0353 (18)	−0.0136 (16)	0.0081 (17)
O5	0.0472 (18)	0.059 (2)	0.0508 (18)	−0.0255 (16)	−0.0102 (14)	0.0031 (15)
N1	0.0381 (18)	0.0404 (19)	0.049 (2)	−0.0148 (15)	−0.0127 (15)	0.0019 (15)
N2	0.065 (3)	0.077 (3)	0.054 (2)	−0.036 (2)	−0.023 (2)	0.010 (2)
C1	0.043 (2)	0.038 (2)	0.044 (2)	−0.0119 (18)	−0.0106 (18)	−0.0002 (17)
C2	0.044 (2)	0.039 (2)	0.041 (2)	−0.0131 (18)	−0.0086 (17)	0.0002 (17)
C3	0.044 (2)	0.055 (3)	0.049 (2)	−0.018 (2)	−0.0040 (19)	−0.001 (2)
C4	0.065 (3)	0.071 (3)	0.037 (2)	−0.028 (3)	−0.006 (2)	−0.001 (2)
C5	0.061 (3)	0.062 (3)	0.045 (2)	−0.026 (2)	−0.014 (2)	0.000 (2)
C6	0.049 (3)	0.061 (3)	0.048 (3)	−0.025 (2)	−0.012 (2)	0.001 (2)
C7	0.047 (2)	0.050 (2)	0.041 (2)	−0.020 (2)	−0.0047 (18)	0.0025 (18)
C8	0.083 (4)	0.106 (5)	0.050 (3)	−0.046 (4)	−0.017 (3)	−0.003 (3)
C9	0.041 (2)	0.047 (2)	0.048 (2)	−0.0174 (19)	−0.0145 (18)	0.0010 (18)
C10	0.054 (3)	0.044 (2)	0.056 (3)	−0.023 (2)	−0.013 (2)	0.003 (2)
C11	0.049 (2)	0.041 (2)	0.051 (3)	−0.016 (2)	−0.011 (2)	0.0079 (19)
C12	0.041 (2)	0.049 (2)	0.038 (2)	−0.0166 (19)	−0.0089 (17)	0.0038 (18)
C13	0.042 (2)	0.041 (2)	0.044 (2)	−0.0156 (18)	−0.0107 (17)	0.0020 (17)
C14	0.044 (2)	0.049 (3)	0.049 (2)	−0.018 (2)	−0.0126 (19)	0.0054 (19)
C15	0.087 (4)	0.084 (4)	0.057 (3)	−0.040 (3)	−0.015 (3)	0.005 (3)
C16	0.109 (6)	0.178 (9)	0.122 (7)	−0.066 (7)	0.029 (5)	−0.069 (7)
C17	0.082 (4)	0.075 (4)	0.080 (4)	−0.032 (3)	−0.040 (3)	0.017 (3)
C18	0.134 (7)	0.086 (5)	0.088 (5)	−0.046 (5)	−0.049 (5)	−0.001 (4)

### Geometric parameters (Å, °)

**Table:**

Co1—O1	2.088 (3)	C7—C6	1.370 (6)
Co1—O1^i^	2.088 (3)	C7—H7	0.9300
Co1—O5	2.121 (3)	C8—H81	1.04 (6)
Co1—O5^i^	2.121 (3)	C9—H9	0.9300
Co1—N1	2.163 (3)	C10—C9	1.377 (6)
Co1—N1^i^	2.163 (3)	C10—C11	1.385 (6)
O1—C1	1.262 (5)	C10—H10	0.9300
O2—C1	1.257 (5)	C11—H11	0.9300
O3—C8	1.193 (7)	C12—C11	1.385 (6)
O4—C14	1.219 (5)	C12—C13	1.378 (6)
O5—H51	0.94 (4)	C13—H13	0.9300
O5—H52	0.92 (2)	C14—N2	1.330 (6)
N1—C9	1.341 (5)	C14—C12	1.511 (6)
N1—C13	1.343 (5)	C15—C16	1.476 (9)
N2—C15	1.473 (7)	C15—H15A	0.9700
N2—C17	1.501 (7)	C15—H15B	0.9700
C2—C1	1.504 (6)	C16—H16A	0.9600
C2—C3	1.391 (6)	C16—H16B	0.9600
C2—C7	1.392 (6)	C16—H16C	0.9600
C3—C4	1.371 (6)	C17—C18	1.463 (8)
C3—H3	0.9300	C17—H17A	0.9700
C4—C5	1.394 (7)	C17—H17B	0.9700
C4—H4	0.9300	C18—H18A	0.9600
C5—C6	1.391 (6)	C18—H18B	0.9600
C5—C8	1.477 (7)	C18—H18C	0.9600
C6—H6	0.9300		
			
O1^i^—Co1—O1	180.00 (17)	O3—C8—C5	126.1 (6)
O1^i^—Co1—O5^i^	88.16 (12)	O3—C8—H81	116 (3)
O1—Co1—O5^i^	91.84 (12)	C5—C8—H81	117 (3)
O1^i^—Co1—O5	91.84 (12)	N1—C9—C10	123.3 (4)
O1—Co1—O5	88.16 (12)	N1—C9—H9	118.3
O1^i^—Co1—N1	91.25 (12)	C10—C9—H9	118.3
O1—Co1—N1	88.75 (12)	C9—C10—C11	118.5 (4)
O1^i^—Co1—N1^i^	88.75 (12)	C9—C10—H10	120.7
O1—Co1—N1^i^	91.25 (12)	C11—C10—H10	120.7
O5^i^—Co1—O5	180.00 (18)	C10—C11—H11	120.6
O5^i^—Co1—N1	93.36 (12)	C12—C11—C10	118.8 (4)
O5—Co1—N1	86.64 (12)	C12—C11—H11	120.6
O5^i^—Co1—N1^i^	86.64 (12)	C11—C12—C14	123.6 (4)
O5—Co1—N1^i^	93.36 (12)	C13—C12—C11	119.1 (4)
N1—Co1—N1^i^	180.0 (2)	C13—C12—C14	116.9 (4)
C1—O1—Co1	127.5 (3)	N1—C13—C12	122.6 (4)
Co1—O5—H51	119 (4)	N1—C13—H13	118.7
Co1—O5—H52	97 (3)	C12—C13—H13	118.7
H52—O5—H51	105 (3)	O4—C14—N2	122.1 (4)
C9—N1—Co1	123.6 (3)	O4—C14—C12	117.9 (4)
C9—N1—C13	117.7 (4)	N2—C14—C12	120.0 (4)
C13—N1—Co1	118.6 (3)	N2—C15—C16	113.8 (6)
C14—N2—C15	125.4 (4)	N2—C15—H15A	108.8
C14—N2—C17	116.9 (4)	N2—C15—H15B	108.8
C15—N2—C17	117.6 (4)	C16—C15—H15A	108.8
O1—C1—C2	116.9 (4)	C16—C15—H15B	108.8
O2—C1—O1	125.1 (4)	H15A—C15—H15B	107.7
O2—C1—C2	118.0 (4)	C15—C16—H16A	109.5
C3—C2—C1	120.0 (4)	C15—C16—H16B	109.5
C3—C2—C7	118.7 (4)	C15—C16—H16C	109.5
C7—C2—C1	121.3 (4)	H16A—C16—H16B	109.5
C2—C3—H3	119.9	H16A—C16—H16C	109.5
C4—C3—C2	120.3 (4)	H16B—C16—H16C	109.5
C4—C3—H3	119.9	N2—C17—H17A	109.4
C3—C4—C5	120.5 (4)	N2—C17—H17B	109.4
C3—C4—H4	119.7	C18—C17—N2	111.3 (5)
C5—C4—H4	119.7	C18—C17—H17A	109.4
C4—C5—C8	119.7 (5)	C18—C17—H17B	109.4
C6—C5—C4	119.5 (4)	H17A—C17—H17B	108.0
C6—C5—C8	120.7 (5)	C17—C18—H18A	109.5
C5—C6—H6	120.2	C17—C18—H18B	109.5
C7—C6—C5	119.5 (4)	C17—C18—H18C	109.5
C7—C6—H6	120.2	H18A—C18—H18B	109.5
C2—C7—H7	119.3	H18A—C18—H18C	109.5
C6—C7—C2	121.4 (4)	H18B—C18—H18C	109.5
C6—C7—H7	119.3		
			
O5^i^—Co1—O1—C1	−11.3 (4)	C1—C2—C3—C4	179.5 (4)
O5—Co1—O1—C1	168.7 (4)	C7—C2—C3—C4	−1.1 (7)
N1—Co1—O1—C1	82.0 (4)	C1—C2—C7—C6	−178.9 (4)
N1^i^—Co1—O1—C1	−98.0 (4)	C3—C2—C7—C6	1.8 (7)
O1^i^—Co1—N1—C9	33.5 (3)	C2—C3—C4—C5	−0.3 (7)
O1—Co1—N1—C9	−146.5 (3)	C3—C4—C5—C6	1.2 (8)
O1^i^—Co1—N1—C13	−148.7 (3)	C3—C4—C5—C8	−179.0 (5)
O1—Co1—N1—C13	31.3 (3)	C4—C5—C6—C7	−0.6 (8)
O5^i^—Co1—N1—C9	−54.7 (3)	C8—C5—C6—C7	179.6 (5)
O5—Co1—N1—C9	125.3 (3)	C4—C5—C8—O3	174.2 (7)
O5^i^—Co1—N1—C13	123.0 (3)	C6—C5—C8—O3	−6.0 (10)
O5—Co1—N1—C13	−57.0 (3)	C2—C7—C6—C5	−1.0 (7)
Co1—O1—C1—O2	27.6 (6)	C11—C10—C9—N1	−0.6 (7)
Co1—O1—C1—C2	−151.1 (3)	C9—C10—C11—C12	1.5 (7)
Co1—N1—C9—C10	177.3 (3)	C13—C12—C11—C10	−1.2 (6)
C13—N1—C9—C10	−0.5 (6)	C14—C12—C11—C10	171.5 (4)
Co1—N1—C13—C12	−177.2 (3)	C11—C12—C13—N1	0.1 (6)
C9—N1—C13—C12	0.7 (6)	C14—C12—C13—N1	−173.1 (4)
C14—N2—C15—C16	109.8 (7)	O4—C14—C12—C11	−114.3 (5)
C17—N2—C15—C16	−70.5 (8)	O4—C14—C12—C13	58.6 (6)
C14—N2—C17—C18	−77.7 (7)	N2—C14—C12—C11	62.5 (6)
C15—N2—C17—C18	102.6 (6)	N2—C14—C12—C13	−124.6 (5)
C3—C2—C1—O1	175.2 (4)	O4—C14—N2—C15	−178.4 (5)
C3—C2—C1—O2	−3.6 (6)	O4—C14—N2—C17	1.9 (7)
C7—C2—C1—O1	−4.1 (6)	C12—C14—N2—C15	5.0 (8)
C7—C2—C1—O2	177.1 (4)	C12—C14—N2—C17	−174.7 (4)

Symmetry codes: (i) −*x*+1, −*y*, −*z*.

### Hydrogen-bond geometry (Å, °)

**Table:**

*D*—H···*A*	*D*—H	H···*A*	*D*···*A*	*D*—H···*A*
O5—H51···O4^ii^	0.94 (4)	1.86 (4)	2.787 (5)	174 (6)
O5—H52···O2^i^	0.92 (2)	1.73 (4)	2.646 (5)	168 (6)

Symmetry codes: (ii) −*x*, −*y*, −*z*; (i) −*x*+1, −*y*, −*z*.
